# A qualitative exploration of what works for migrant adolescents in transcultural psychotherapy: perceptions of adolescents, their parents, and their therapists

**DOI:** 10.1186/s12888-020-02970-w

**Published:** 2020-11-26

**Authors:** Léa Grau, Emilie Carretier, Marie-Rose Moro, Anne Revah-Levy, Jordan Sibeoni, Jonathan Lachal

**Affiliations:** 1 UFR des Lettres, des Sciences de l’Homme et des Sociétés Unité Transversale de Recherche Psychogenèse et Psychopathologie, UTRPP EA4403, Univ. Paris 13, F-93430 Villetaneuse, France; 2 AP-HP, Hôpital Cochin, Maison de Solenn, 97 Boulevard de Port Royal, 75014 Paris, France; 3 Université Paris-Saclay, UVSQ, Inserm, CESP, Team DevPsy, F-94807 Villejuif, France; 4 Université de Paris, PCPP, F-92100 Boulogne-Billancourt, France; 5 Service Universitaire de Psychiatrie de l’Adolescent, Centre Hospitalier d’Argenteuil, F95100 Argenteuil, France; 6 ECSTRRA Team, UMR-1153, Inserm, Université de Paris, Paris, France

**Keywords:** Transcultural psychotherapy, Migrant adolescents, Qualitative research, Evaluation of psychotherapy, Self-disclosure

## Abstract

**Background:**

Migrant adolescents are at a higher risk than their native-born counterparts of psychiatric disorders, and their care is a public health issue. In France, transcultural psychotherapy is a treatment provided by a group of therapists designed to meet the specific needs of these patients when usual care appears ineffective. The objective of this study was to explore the therapeutic elements at work in transcultural psychotherapy.

**Methods:**

We conducted a qualitative study crossing the perspectives of adolescents receiving transcultural psychotherapy, their parents, their first-line therapist (FLT), and the transcultural therapists. The families were chosen by purposive sampling. Data were collected during semi-structured individual (for FLTs) and group (families and transcultural therapists) interviews that explored the therapeutic elements involved and effective in transcultural psychotherapy. We used *interpretative phenomenological analysis* (IPA) to examine the data. In all, 44 participants were questioned: three adolescents (2 girls and 1 boy, all aged 18 to 21 years) and their parents (3 mothers and 1 father), three FLTs (2 child psychiatrists and 1 psychologist), and the 34 therapists participating in the three transcultural psychotherapy groups.

**Results:**

The analysis uncovered three themes: (1) the perceived effectiveness of the group’s functioning; (2) the recounting of the individual, family, and cultural history to allow for complexity and nuance; and (3) the personal investment by therapists, made possible by the group.

**Conclusions:**

Our results show some therapeutic elements at work in transcultural psychotherapy that enable it to meet the particular needs of some migrant adolescents that are unmet in standard therapy. Continuing to study transcultural psychotherapy and assess its effectiveness is essential for promoting and optimizing psychiatric care for migrant adolescents.

## Background

Migration was a reality for more than 70 million people in 2019 according to the United Nations High Commission for Refugees, and the number of migrant children, who either made the journey themselves (first generation) or were born in the host country with at least one parent born abroad (second-generation migrants), continues to grow in Western Europe [1, 2].

Migrant adolescents are especially vulnerable, to a greater degree than their host country counterparts [1, 3, 4], and this vulnerability is only partially explained by socioeconomic factors [5]. The clinical expression of psychiatric disorders is strongly influenced by its cultural context. This in turn induces a risk of misdiagnosis and misunderstanding by healthcare professionals [6, 7]. Numerous barriers result in the underuse of mental health services by migrant families, regardless of the host country and the organization of its healthcare system [8]. Psychiatric care is often longer and less effective than in the general population, with a greater risk that treatment will fail [9] and the disorder become chronic [3].

Numerous responses have been proposed to this growing public health issue, depending on the mental health care context and on each country’s history and pattern of migration [10, 11]. Some countries, such as the United States, have promoted ethnic matching between therapists and patients, but most countries have focused on increasing cultural competence of professionals by training and supervision and developed models relying on interpreters and cultural mediators [12–14]. Several interventions for children and adolescents with a migration background who suffer from depression or post-traumatic stress disorder — such as eye movement desensitization and reprocessing, narrative exposure therapy, trauma-focused cognitive behavioral therapy, and multifamily therapy — have shown promising results [15–18].

Psychiatrists in France have developed a complete second-line psychotherapeutic method: transcultural psychotherapy [19]. Patients, with their families, are referred for this care by their first-line therapist (FLT) when standard treatment fails to enable, for example, a diagnosis or the establishment of a good therapeutic alliance. This method is based on the paradigm of psychic universality — that psychic functioning is the same for all and that we must give the same status to all human beings and to their psychic and cultural production, their ways of living and thinking. It also relies on Devereux’s technique of complementarity: complementary and nonsimultaneous analysis of the material from sessions, from two scientific perspectives: anthropology and psychoanalysis [20–22]. The clinical approach relies on elements from systemic and psychoanalytic family therapy, narrative therapy, and cultural mediation. The patient, accompanied by his or her family, is received by a group of therapists (psychiatrists, psychologists, and nurses) of diverse cultural origins, trained in transcultural psychotherapy; the group also includes trainees. In contrast to systemic therapy, the therapists are physically present in the group and explain their ideas and remarks in front of the family. In traditional societies, individuals are thought to be in constant interaction with the group to which they belong, and illness is seen as an event concerning not only the sick individual, but also the family and the group. This concept explains the importance of the group in some care situations. It is a flexible system — a variable-geometry setting — and its size (around 10 therapists) can be adapted to the clinical situation (such as trauma or unaccompanied minors) and to the family’s cultural origin. A principal therapist leads the sessions and calls upon the other therapists to speak, asking them for comments, representations, interpretations, or metaphors. The indirect communication, in which the co-therapists speak only to the principal therapist, who can rephrase some remarks to the patients, promotes the group’s holding (in the Winnicottian sense of the word). An interpreter in the family’s native language is generally present, thus promoting the use of that language and enabling family members to express their emotions and personal thoughts fully. This way, the group becomes a transitional space between the culture of origin and that of the host country and allows the establishment of a dialogue about the disease, its causes, and its treatments. The aims of transcultural psychotherapy are multiple: promoting the therapeutic alliance, refining the diagnosis, using culture as a therapeutic lever and thus supporting a reflexive process and elaboration within the family aimed at reducing symptoms and improving family functioning. Sessions last about an hour and a half and take place every 6 weeks. The first line treatment — psychiatric care using either or both of medication and psychotherapy — continues throughout the transcultural psychotherapy, which the FLT is also asked to attend.

The evaluation of psychotherapies is complex, from not only an epistemological but also a methodological perspective. Most studies that have sought to compare the effects of different types of psychotherapy show the importance of common curative elements (such as empathy or the therapeutic alliance), while the specific psychotherapeutic techniques may play a minor role in the outcome of the therapy [23–25]. It has been demonstrated in both children and adolescents that a good therapeutic alliance with both the patient [26] and the parents [27] promotes the effectiveness of the therapy. Yet, establishing a good therapeutic alliance is one of the main challenges in mental health care for migrant children and adolescents.

Numerous clinical studies have enabled the development of various theoretical hypotheses about the therapeutic factors underlying the framework and techniques of transcultural psychotherapy [22, 28, 29], and studies are underway to document its effectiveness [30]. These studies insist on the role of the group as a representation or figuration of cultural alterity, and the therapeutic work of *metissage* (mixing or hybridizing the cultures of the home and host countries). Nonetheless, no study has examined simultaneously the points of view of both patients and therapists about these therapeutic elements, although their experience is central in treatment [31, 32] and in research [33]. Qualitative methods are especially appropriate when the objective is to explore the point of view or experience of participants about health care or about the elements of the therapy that work, that are perceived as effective [34–36]. They have previously been used to explore the elements or processes of change [37], but no study has combined the points of view of patients and therapists to analyze the elements of change at work in psychotherapy involving migrants.

Our study is an exploratory stage of a broader project intended to assess transcultural psychotherapy qualitatively and quantitatively. Our objective here is to explore qualitatively the therapeutic elements at work in transcultural psychotherapy for adolescents, and we started by collecting the experiences of patients and their families, the transcultural psychotherapists, and their FLTs. Putting into perspective the experiences of the different participants should improve our understanding of the elements of transcultural psychiatry that are effective in treating patients, that is, that help patients get better. This study complements another exploratory study that examined the experience of transcultural psychotherapy and its acceptability from the perspective of migrant adolescents and their families [38].

## Methods

This is an exploratory qualitative study approved by the Research Ethics Board of Sud Mediterranean IV.

### Population and sampling

The population comprises the adolescent patients and their families referred for transcultural psychotherapy at a mental health center for adolescents in Paris, their FLTs, and the transcultural psychotherapists.

- The inclusion criteria for the adolescents were: an adolescent migrant (first or second generation) aged 13–21 years, with any psychiatric disorder, seen for transcultural psychotherapy for a period long enough (3 sessions or around 4 months of follow-up) to allow exploration of these therapeutic elements, and whose FLT was still currently treating the patient.
- All transcultural therapists involved with these adolescents were included in the study.

Three families were chosen by purposive sampling [36, 39]; in this case, that means that we selected them because transcultural therapists considered the sessions beneficial for them. They were thus likely to provide the most information about the therapeutic elements at work in transcultural psychotherapy. In all, 44 participants were questioned during 10 interviews: 3 teens (2 girls and 1 boy) and their parents (3 mothers and 1 father), three FLTs (1 psychologist and 2 child psychiatrists), and each member of the three transcultural psychotherapist groups (34 therapists) (Table 1).

**Table 1:** Characteristics of participants

Adolescent	Age	Country of origin	Generation of migration	Time in the host country	Symptoms	Number of transcultural sessions conducted	Family members questioned	Interpreter during the interview	First-line therapist	Transcultural therapists
P1	20–22	Sri Lanka	Second	From birth	Borderline personality disorder	8	P1 + her mother	Yes	Psychologist	Transcultural group created in 2008: Principal therapist: psychiatrist. 7 co-therapists: 1 psychiatrist, 4 psychologists, 1 resident in psychiatry, 1 nurse. 3 trainees.
P2	17–19	China	Second	From birth	Anorexia nervosa	35	P2 + her father and her mother	No	Child psychiatrist	Transcultural group created in 2008: Principal therapist: psychiatrist. 5 co-therapists: 1 psychiatrist, 3 psychologists, 1 resident in psychiatry. 9 trainees
P3	20–22	Cameroon	First	For 8 years	Atypical depression	18	P3 + her mother	No	Child psychiatrist	Transcultural group created in 2012: Principal therapist: psychiatrist. 4 co-therapists: 2 psychiatrists, 1 nurse, 1 resident in psychiatry. 3 trainees

### Data collection

The study was first explained to the group of transcultural therapists. The families were informed about this research either by the principal therapist at the end of a session, or by the referral nurse in a telephone call. A meeting was set up, during which adolescents and family members received clear oral and written information and provided written consent, in the presence of an interpreter. The interviewer had no relationship with the patients or their families before the study began. FLTs of the participating adolescents were informed of the study by email or telephone. They too received clear information and provided written consent to participate, as did the transcultural therapists. The following research interviews were organized: one with each adolescent and their parents, one with each FLT, and one with each of the three transcultural psychotherapy groups, as well as an additional individual interview with a transcultural therapist who missed the group interview.

The data were collected in semi-structured interviews, each about an hour, conducted by two researchers (LG and EC). The research team (specialists in transcultural care and in qualitative methods) developed the interview guides during meetings and adapted them progressively during the study (Table 2).

**Table 2:** Interview guide

Families	First-line therapists (FLTs)	Transcultural therapists
1 Can you tell me something about your journey to this transcultural psychotherapy program?	How did you come to propose this transcultural consultation to this family? What factors led you to think it was indicated?	What factors do you think led the FLT to propose treatment with transcultural psychotherapy?
2 Do you remember what you felt during the first session and then the following? Can you tell me your memories of these sessions that affected you most? Is there a moment during the session that was most important to you?	Do you remember what you felt during the first session and then the following? How do you think that the family experienced these sessions? Can you tell me your most vivid memories of these sessions?	How did the first session go? And afterwards? What are your most vivid memories of this therapy?
3 What effects did the transcultural psychotherapy have (on you, your child, your family)? Can you describe them? What changes occurred?	What effects did the transcultural psychotherapy have on your patient? On the family? What changes did you observe?	What effects did the transcultural psychotherapy have on the patient? On the patient’s family? What changes did you observe?
4 What seemed useful to you in transcultural psychotherapy to make progress in the problems that existed at the start? What is it that helped you? Was there a moment that seemed decisive to you in this treatment? Can you tell me about it?	What do you think are the elements of transcultural psychotherapy that had a positive effect, that enabled changes? Was there a moment that seemed decisive to you in this treatment? Can you tell me about it?	What do you think are the elements of transcultural psychotherapy that had a positive effect, that enabled changes? Was there a moment that seemed decisive to you in this treatment? Can you tell me about it?

The family interviews included all of the family members who agreed to participate. They took place in French or with an interpreter present when necessary. The second interview was with the FLT. The third interview, which was really more of a focus group, included the entire group of transcultural therapists seeing that patient, so that they could interact and compare and contrast their points of view of the clinical situation. All of the interviews were audio recorded (digitally), anonymized, and transcribed word for word.

### Data analysis

The interviews were analyzed by using *interpretative phenomenological analysis* (IPA) [40], a method used to understand how people subjectively perceive an experience and how they make sense of it [41].

Word and Excel software facilitated several repeated readings of the interviews and then their annotation and coding to identify the emerging themes. Connections between these emerging themes were sought and used to link them. Each interview was analyzed independently. The results were then compared to identify recurrent themes but also to integrate the new emerging elements. The interviews with the families were analyzed first, then the FLT interviews, and finally the focus groups of the transcultural therapy groups. This separation was intended to prevent the theoretical elements expressed by the therapists with expertise in transcultural psychotherapy from affecting the interpretation of the other interviews. The results as a whole were then compared to construct a complete thematic description of the therapeutic elements identified by the participants.

To ensure the validity of our research, the interviews were analyzed independently by three researchers (LG, JL, and JS) and the divergences discussed in research team meetings.

## Results

The analysis of the data uncovered three superordinate themes: (1) the perceived effectiveness of the group’s functioning; (2) the recounting of the individual, family, and cultural history to allow for complexity and nuance; and (3) the personal investment by therapists, made possible by the group.

### Perceived effectiveness of the group’s functioning

#### Providing a safe space

The configuration of the transcultural group allowed patients to find a place that seemed more familiar to them, a place “appropriate for them to blossom”. One adolescent, for example, compared it to his feelings when he visits the embassy of his country of origin.

*"For example if I go … to the embassy of Cameroon, we're in France, yes but … I'm going to do the kind of thing a Cameroonian would do. So it's that kind of situation that I thought about … during the sessions. … I think that it's good for the patient to feel they're in an environment that is appropriate for them so that they can blossom." P3.*

The families perceived the transcultural group as a safe space that allowed them to sustain a discourse, to speak, including about personal subjects. In this environment perceived as more tolerant, they felt they were heard and taken seriously, without preexisting prejudices.

*"Little by little, we start to touch on sensitive issues. Little by little, we talk … Thanks to these transcultural sessions, now we talk about fundamental, very serious things." P2's father*

*"They told us that they don't have prejudices or bias. …They take into account that some cultures have different practices than their own. …So we could talk about things freely, it wasn't shocking." P3's mother.*

The FLTs also described this aspect. The transcultural therapists mentioned empathetic and compassionate listening.

*"I think they were glad to find people … who could listen and react with a system of representations closer to theirs. A much more open approach and therefore tolerant." FLT (for P3).*

*"I think that this compassionate and empathetic listening that we were able to provide…" Transcultural co-therapist (for P3).*

#### Carrying and containing

The adolescents and their parents reported that they felt reassured and surrounded within the group and that this feeling enabled them to channel some emotions. The group’s ability to contain anger has been emphasized.

*"Because it is structured here, there are doctors and everything, so as a result, anger can come out, it's supervised in fact." P1*

*"It was important to be in a setting where this anger could be expressed and to be able to channel all these emotions a little. There was something in him, it needed to explode, and it was better that it explodes there." P3's mother*

The transcultural therapists identified a group portage, or carrying, function that they compared to a mother carrying her child. As P1’s principal transcultural therapist pointed out*, “[s]he needed to recenter, and there [in the group] this work assumes* a *great capacity for holding, and I think that it was also possible because the maternal carriage was potentiated by that of the group.”*

Finally, beyond the group dimension, the transcultural therapists underlined that use of the native language also performs a carriage function. One of P1’s transcultural co-therapists pointed out that “[i]t *was done by the group but it was done by the language. The fact that we could move from one language to another like that allowed him to be cradled in both languages.”*

#### Building the therapeutic alliance

The group was invested simultaneously as a group and as a set of individuals. The families and FLTs underlined the different kinds of therapeutic alliances possible with the different therapists in the group. One of the adolescents remembered her reaction a day when the principal therapist, who was absent, was replaced by a co-therapist:

*"As I knew that it was a therapist who was taking the place of the [usual principal therapist], well, so I had the impression that he was a little bit representing all of the other therapists. I have the memory of a better emotion, feeling." P2*

The integration of the FLT and the interpreter into the group enabled further support of the adolescent and the family, providing continuity with the exterior, with the culture. FLTs sometimes served as the “spokesperson” for a patient, allowing them to prepare the transcultural sessions or to talk about them again in individual therapy. P1’s FLT noted that *“even in the waiting room. I was arriving, I was trying to arrive early, she was relieved to see me arrive.”*

According to the transcultural therapists, when it was difficult to establish an alliance with the adolescent, a connection with the parents made it possible to support the family and produce a treatment effect. The group method also enabled the patient, by the diffraction of the transfer and of traumatic elements, to connect to some therapists more than others.

*"No matter where the children were, they came. But we held on to the parents and by doing that, we held on to the children." P2's principal transcultural therapist.*

"*And what can make her bond to you [the principal therapist] massively like that, it's also the fact that we are a group. That is, she can allow herself to really use a second mother, for real... but because she can diffract the rest of feelings and thus diffract the other parts of herself on the group." One of P1's transcultural co-therapists.*

### The recounting of the individual, family, and cultural history to allow for complexity and nuance

#### Supporting weakened parenting functions and working on family conflicts

Work by and within the family took place during transcultural sessions and dealt especially with family conflicts and issues of communication and understanding between parents and adolescents. According to the families, it made it easier to consider in detail the conflicts related to the culture, especially when they were trying to make sense of the disease.

*"When he got sick, that's it, the origin of his disease, it comes from [a spell cast by that marabout]. It's a little the origin of the conflicts and everything." P3's mother.*

This work facilitated transmission and communication between the generations.

*"Finally I found also that something in my mother had changed since the sessions we had and everything, it's that she understands my problems a little more. While before she was more blaming me…, now she's listening more to what's wrong." P1.*

This space let parents mention their difficulties and sense of isolation and especially to feel valued for their skills. P3’s mother noted: “*they congratulated me, they asked me where I got the strength to keep going, with everything that was happening.”*

The interpreter gave the parents the possibility of expressing themselves in their own language and provided the adolescents with the opportunity to not have to be the interpreter and to be replaced in the “right place”.

*"All my life…I've been the interpreter! It's bothered me to do the two things at the same time! And when we got here in fact, what helped me it's that there was someone else who took over that role [of interpreter]. Finally each of us had our own role. Everyone had their own place!" P1.*

The interpreter also played an essential role of third person and mediator. Interpreting made it possible to rephrase, complexify, and qualify words when addressing conflicts.

*"At the beginning I didn't understand, I said to myself: 'but she's translating it wrong, she doesn't understand, and everything!' And in fact it was precisely that she was translating with another meaning. So I understood that you can understand two different thing with one person who's talking the same language, even." P1.*

For the FLTs, the transcultural group made it possible to address the conflicts from a different angle, to take the cultural distance into account, and to avoid situations of misunderstanding and confrontation between the family and the professionals. According to P3’s *FLT, “Family conflicts should be treated some way other than by the threat of a report or a warning [to social services], that is, not as something to be judged or even punished.”*

The transcultural therapists also stressed that empowering the parents helps the entire family. One of P1’s transcultural co-therapists noted that “*for me, the group also helped the mother to be able to like herself a little more. (…) She really feels that she has something valuable to give her daughter.”*

#### Telling the family history and allowing *métissage* and a way out of the splitting

According to the adolescents, hearing their parents’ story and journey during transcultural therapy sessions let them discover or rediscover their family history, understand where they came from and how they could find their place in this family.

*"The history of my parents, and of everything there is beyond… that's part of my identity. Finally it isn't me, but I'm a continuation of it. And I think that I've succeeded in raising [the question of my origins] also by what happens here." P1.*

Transcultural therapists described the transcultural therapy group as a “*neutral territory between two cultures*”. For one of the adolescents interviewed, the question about *métissage* or mixing two cultures seemed to have been central to her management because not only she but also her FLT and the transcultural therapists all mentioned it. This *métissage* allowed her to reaffiliate with her culture of origin and with her family and to calm down.

*"I like it a lot because I've learned here that I am a 'métisse, culturally speaking,' I really like this expression. …I just needed to find a happy medium between two cultures that are very different; I didn't have to choose one culture or another, I bridge them, in fact. Between these different traditions, beliefs and all that." P1.*

Interpreting supported this work of *métissage* and allowed patients to hear their mother tongue positively. For the teens, the interpreter could represent a role model of successful blending between the two cultures. P1’s transcultural co-therapist noted: “*I think that she had needed to hear her language in another way, in something positive, that circulates. And also to reestablish the emotional aspect of her language.”*

#### Focusing on the patient and showing how to make connections and enable therapeutic work

Telling the story is often blocked by a knot that intertwines individual, family, and cultural elements. For some participants, it was necessary to pay less attention to some aspects of the culture or the disease history to focus on the adolescents and offer them a space to become the protagonist of their own story.

The FLT who referred P3 for a family conflict described such a situation. The familial conflict focused on accusations of witchcraft (cultural etiologies), which followed a somatic illness with major physical after-effects for the youth. After a while, the transcultural group chose to shift the topic away from these sequelae, focusing instead on his issues of adolescence and especially his search for autonomy. P3’s FLT described this work as a *“perspective, for sure, that’s further away from the hospital and from medicine than mine.”* The transcultural therapists described this as an attempt “*to downplay what leads to the disorder*”, which sometimes involves not “*focusing on the disease*” or even on the cultural elements. They insisted on the need for management that is adapted to each clinical situation. According to one of P3’s transcultural co-therapists, *“We finally said that what was going to help P3 was to treat him more like an adolescent. It’s true that we’ve also worked a lot on autonomy. In something sort of commonplace.”*

The adolescents described the transcultural group as a place that enabled them to understand and to reappropriate things, to “make connections”, especially between the transcultural psychotherapy and the individual follow-up.

*"Yes I think that I understand some things anyway. I think that it plays a role but together with all the other treatments I have. I make connections with the different types of therapy I have, I deduce things from them." P2.*

The FLTs pointed out that the transcultural program deepened perceptions of some issues. One of the psychologists thus explained that even when the subjects addressed had already been worked on in individual therapy, the transcultural psychotherapy gave it greater depth. P1’s FLT said, “*because it gives another dimension, in fact. Yes, it’s another way to do this, another way to empower, the ‘ethnopsy’ consultations.”*

The transcultural therapists mentioned a work of translation and “*making the implicit explicit”,* which helps patients to make these connections.

### Personal investment by therapists, made possible by the group

#### Therapists as figures to identify with

The adolescents and their parents explained that they were sensitive to the therapists’ investment. The involvement of a group that is available and “takes the time necessary” was stressed several times.

*"It's a group of psychologists that spends much more time than the psychologists…you see every day. It's a group, they take much more time to talk with the patient…No one takes this time...Who is going to take the time to listen to you like that?" P3.*

The transcultural therapists described a group investment — but also individual. Each represented a role model, back-up support, especially for the parents. The group made it possible for each therapist to be invested and to “lend” his or her representations to the parents for help them in the therapeutic work. The diversity of the group (age, gender, and culture) supported this process. One male therapist (in a group with a majority of women) thus stated his position in relation to a father:

*"I was defending the father, a migrant … Perhaps we needed to get between the parents a little, to reinforce the father's position as 'the father'… to support him, back him up." One of P2's* transcultural co-therapists*.*

#### The cultural diversity of the group enables multiple discourses

The cultural diversity within the group of therapists and the fact that it was used as a therapeutic element were underlined by the teens and their parents. They could thus rely on the therapists’ experience and culture. The cultural diversity of the group enabled access to different perceptions, different ways of looking, and helped them to understand some things better.

*"So, it's sort of like at the shrink, but with one thing extra, that at the end I'm going to have different points of view to help me put things in perspective." P1.*

*“I’m going to have different points of view, from doctors of course but from different cultures, which are going to help me to put things into perspective … There are lots of different cultures that are going to see it differently and it’s a question of perception in fact… Depending on where I am and the culture I grew up in, well, I'll see things differently.” P1*

*"There was one co-therapist for example who had said that her parents also had never said to her, 'I love you'. With words. It was a Japanese woman. That really made an impression on me, that she said that." P1's mother (through the interpreter).*

For the transcultural therapists, this was a central element of the program, which gave patients and their families access to other representations, allowed them to experiment with other positions. P1’s principal transcultural therapist described it: *“And then she can experiment, like that, see ‘what happens if I put myself on that side, what happens if I put myself on the other side’…”.*

The cultural diversity of the therapists and the interpreter with the same culture offered the family the possibility of using elements of their own culture, of their own representation of the disease and its care. P3’s FLT pointed out that, contrary to common practice, the cultural difference was used for the treatment rather than viewed as an obstacle.

*"The fact that there were lots of professionals … they understood the things that were related to witchcraft." P3's mother.*

*"Here cultural difference or otherness is experienced too often as being the problem in fact. While over there [at the transcultural program] they use it … But also the culture of the co-therapists." P3's FLT.*

#### Sharing the therapists’ personal relation to the culture

The disclosure by the therapists of elements of their history and their personal relation to culture while offering interpretations and metaphors might surprise some families, but they awaited this moment of the session impatiently.

*"In fact, what I like a lot in these sessions, it's really the moment when we move to the interpretations. Each one says truly their own metaphor, their idea, and all, and I find that is actually wonderful." P1.*

*"You really can use your culture to treat someone. There, when shrinks are still essentially trained to...to say nothing about themselves ... well, you know what I mean." P3's FLT.*

The group dynamics, the supervision, and the possibility of developing each therapist’s cultural countertransference together allowed the individual investment and the disclosure of their own culture. The trust between the therapists permitted both the wealth of the elaboration and the verbalization of suggestions that were sometimes contradictory.

*" Without going beyond that because outside of a transcultural group, that could have been a problem, the diversity of the countertransferences is what made it possible. One of the essential qualities of the group, I think." P2's transcultural co-therapist.*

## Discussion

This qualitative study of families seen in transcultural psychotherapy, their FLTs, and the therapists in the transcultural groups is the first to put into perspective the experience of the three groups of participants about what works in this psychotherapy.

Our results present both factors common to many forms of psychotherapy and other factors that are much more specific to transcultural psychotherapy. We mentioned in the introduction the importance of the therapeutic alliance as a common factor. Yet, the construction of this alliance with migrant adolescents is a challenge that is often the reason the family is referred for transcultural psychotherapy. The high attendance rate at sessions — more than 80% — suggests that this second-line therapy is rising to this challenge [42].

Our study points out specific therapeutic elements at work in this method that can enable the building of the therapeutic alliance, even in complicated clinical and family situations. These elements are related to the group’s functioning as a safe and containing place and to the work with and within the family, which enables parenting support and may uncover explanations of cultural and family history and conflicts. The group thus sustains the processes of identity construction and of *métissage* between cultures. The interpreter also plays a central role in providing the feeling of a safe place, in containing the family through language, in supporting parental functions and in offering a role model to the adolescents.

The therapists’ personal investment also appears in participants’ discourse as an important specific factor. Transcultural therapists are encouraged to connect personally and to share elements of their own culture and personal history. Although different authors have theorized that this practice, which can be compared to self-disclosure [43], plays a role in transcultural psychotherapy [19, 22], this is the first time that it has appeared as an important element of the therapeutic process, according to the therapists but also the families.

The practice of self-disclosure by therapists is controversial in the psychiatric literature [43]. Therapists’ opinions on this subject are often influenced by their theoretical orientation [44]. Self-disclosure can concern facts of a personal or countertransference-related nature. Some consider that the therapist’s self-disclosure creates a risk of moving the therapeutic work outside of the patient. Moreover, patients may feel threatened by access to the therapist’s limitations or vulnerability [43]. For other, especially humanist theoreticians, this practice may on the contrary make the therapist “more human” and the therapeutic relationship less asymmetric. It may promote the therapeutic alliance, especially with adolescents, and may help normalize the patient’s experience [43–47]. When used in a measured and appropriate manner, self-disclosure may strengthen the therapeutic relationship and support the patient’s work in therapy.

Self-disclosure in transcultural psychotherapy is neither improvised nor sudden; rather it is itself a psychotherapeutic technique and designed as such. In this respect, this form of psychotherapy departs from the psychoanalytical tradition.

This concept of self-disclosure seems original and relevant and offers an interesting reading grid for our results as it sustains several therapeutic elements described by the participants.

First, it allows the establishment of a less asymmetric and more balanced relationship between therapists and patients. In that way, it moves away from the more classic psychotherapeutic methods, in which migrant patients are by definition in an asymmetric relationship. They belong to a minority culture, after all, and are seen in facilities affiliated with the majority culture. This better balance, made possible by the therapists’ cultural diversity and self-disclosure, helps to create a treatment space that is safe, empathetic, and compassionate for the patients, as described by the participants of this study. It facilitates the therapeutic alliance. It thus completes the therapeutic factors common to all group psychotherapies, such as Winnicott’s *holding* [48], Bion’s containment [49], or Kaës’s concept of the “group psychic apparatus” [50].

Second, the cultural diversity of the group and the practice of self-disclosure play a role in the establishment of a multiperspective narrative. This in turn supports the emergence of self-narratives by patients and their families [51]. This disclosure by the therapists encourages the family to participate in a dynamic exchange with them and in the co-construction of a narrative. Our results point out that this narration takes place at the individual, family, but also transcultural levels. It enables work on transmission and lets the adolescents re-enroll in their filiation and their affiliations [29, 51]. It supports their process of identity construction, especially in adolescence. The therapists’ self-disclosure of elements of their culture, their professional and personal experience, enables the emergence of the patients’ discourse about their own culture and about stories difficult to share individually. Families explained that they could develop etiological theories, bit by bit, with the group, that is, cultural theories that let them make sense of the disease and their distress, “make some sense of the senseless (‘donner du sens à l’insensé’)” [52]. These stories sometimes develop spontaneously but sometimes they emerge “in response” to images and interpretations proposed by the therapists, in a dynamic of new exchange and co-construction. The patients thus weave associations between different universes and develop their own treatment strategies. The presence of an interpreter and the possibility of going back and forth between the languages supports this process; the culture serves as a wellspring of creativity.

Third, this practice of self-disclosure sustains the embodiment of otherness. In the transcultural group, the group itself and the multiplicity of therapists embody otherness (as in all types of group psychotherapies) [53]. But it is also embodied by the therapists’ cultural diversity. It may be visible or invisible but it is conveyed above all else by the contents of the discourse. The therapists bring with them their cultures, their personal histories, and their experiences. They “give” these to the families. Families of this study were sensitive to this dimension of the gift and of their disclosure. This embodiment of otherness opens up other perspectives, other representations of what the family is going through. It offers a multitude of hypotheses for a problem, as underlined by P1 who explained that listening the points of view of therapists from different cultures helped her to put things into perspective. It supports a reflexive process, of associations and elaboration and helps to move towards more nuanced, more ambivalent, and more mixed representations [28]. It offers the possibility of addressing and dealing with family conflicts and completes the tools borrowed, for example, from family systems theory, such as circular questioning [54]. It is possible here to draw a parallel with learning how to decenter — a transcultural concept developed from Piaget’s *decentration*, involving the need to “not interpret the unknown in terms of the known” [22] — which is part of the training of all transcultural therapists. By “lending” their representations to the families, the therapists enable them to decenter themselves. The families learn to reappropriate the therapists’ representations and are then able to choose and construct the meaning that they give to what they are experiencing.

### Strengths and limitations of this study

The therapeutic factors of transcultural psychotherapy have been theorized in the literature. This study is nonetheless the first to have explored the therapeutic elements at work in transcultural psychotherapy of migrant teens, by comparing the accounts of the patients, their families, their FLTs, and their transcultural therapists. Although some results are related to factors common to various types of psychotherapy, others on the contrary are very specific to transcultural psychotherapy. In a context where psychological care for migrant children and adolescents is a public health issue, transcultural psychotherapy makes it possible to meet the specific needs of this patient population. This study supports the idea that as second-line treatment, transcultural psychotherapy represents an alternative to the standard management that is often ineffective, long, and expensive.

However, we must highlight some limitations of the study. First, even if we chose the participants by *purposive sampling*, the fact that we have included only three families — and three FLTs — limits the generalizability of our results. Moreover, in most studies comparing the perspectives of patients and therapists on the subject of a psychotherapeutic method [55], or those of children and parents during the psychotherapy of children [56], the themes emerging from the interviews differ notably between the groups of participants. In our study, on the contrary, the principal elements underlined as helpful for treatment by the teens and their parents are quite similar to those mentioned by the FLTs and the transcultural group therapists. The quality of the therapeutic alliance and the effectiveness of the psychotherapy are correlated with the degree of convergence of the perspectives of the patients and therapist [57]. Moreover, the small number of families included and our choice to include specifically the families for whom the therapists had judged transcultural psychotherapy beneficial are hypotheses that might explain this convergence of perspectives. A third limitation, which we had anticipated, involves the fact that the transcultural therapists were often tempted to mention the theoretical elements of this transcultural psychotherapy rather than their experience of the clinical situations studied. We nonetheless took care to analyze the material from each group of participants independently to limit the dissemination of these theoretical elements. Finally, it is important to recall that this study took place in France, within the framework of the French school of transcultural psychotherapy and that the results cannot be generalized to all of the methods of care provided for migrant populations across the world.

## Conclusions

### Important findings and practical implications

This study identifies several therapeutic elements at work in transcultural psychotherapy of migrant teens. Some of them are related to factors common to various types of psychotherapy — such as the carrying and containing effects of the group. But others, on the contrary, are very specific to transcultural psychotherapy such as using culture as a therapeutic lever and self-disclosure as a way of co-constructing a narrative.

Although transcultural psychotherapy is an expensive process requiring numerous therapists, it appears to meet the needs of migrant adolescents and to enable the building of therapeutic alliance even when various obstacles are present. It can avoid years of expensive and ineffective treatments. Our study suggests that FLTs should refer families more often when they face difficulties with migrant families. As a second-line treatment, transcultural psychotherapy should be more commonly accessible and more therapists should be trained in this technique.

### Perspectives for research

These results must be confirmed by other qualitative but also quantitative studies to assess the effectiveness of transcultural psychotherapy and to be able to promote this psychotherapeutic method. This study was an exploratory stage of a broader and national project intended to meet this objective with mixed methods including qualitative and quantitative research [30].

## Data Availability

The data are not publicly available due to privacy restrictions but are available from the corresponding author on reasonable request.

## Abbreviations

IPA: Interpretatvie Phenomenological Analysis
FLT: First-line therapist

## References

[1] Ceri V, Özlü-Erkilic Z, Özer Ü, Kadak T, Winkler D, Dogangün B (2017). Mental health problems of second generation children and adolescents with migration background. Int J Psychiatry Clin Pract.

[2] Naissances selon la nationalité et le pays de naissance des parents | Insee. 2018 [cité 17 mai 2020]. Disponible sur: https://www.insee.fr/fr/statistiques/2381382.

[3] Belhadj Kouider E, Koglin U, Petermann F (2014). Emotional and behavioral problems in migrant children and adolescents in Europe: a systematic review. Eur Child Adolesc Psychiatry.

[4] de Anstiss H, Ziaian T, Procter N, Warland J, Baghurst P (2009). Help-seeking for mental health problems in young refugees: a review of the literature with implications for policy, practice, and research. Transcult Psychiatry.

[5] Sirin SR, Ryce P, Gupta T, Rogers-Sirin L (2013). The role of acculturative stress on mental health symptoms for immigrant adolescents: a longitudinal investigation. Dev Psychol.

[6] Kirmayer L, Fung K, Rousseau C, Lo H-T, Menzies P, Guzder J (2012). Guidelines for training in cultural psychiatry-position paper. Can J Psychiatry.

[7] Kleinman AM (1977). Depression, somatization and the “new cross-cultural psychiatry”. Soc Sci Med.

[8] Nadeau L, Jaimes A, Johnson-Lafleur J, Rousseau C (2017). Perspectives of migrant youth, parents and clinicians on community-based mental health services: negotiating safe pathways. J Child Fam Stud.

[9] Cummings JR, Druss BG (2011). Racial/ethnic differences in mental health service use among adolescents with major depression. J Am Acad Child Adolesc Psychiatry.

[10] Carballeira Carrera L, Lévesque-Daniel S, Radjack R, Moro M, Lachal J (2019). Clinical approaches to cultural diversity in mental health care and specificities of French transcultural consultations.

[11] Kirmayer L, Guzder J, Rousseau C, éditeurs. Cultural consultation: encountering the other in mental health care. New York: Springer-Verlag; 2014 [cité 8 mai 2019]. (International and cultural psychology). Disponible sur: https://www.springer.com/us/book/9781461476146.

[12] Kirmayer LJ, Minas H (2000). The future of cultural psychiatry: an international perspective. Can J Psychiatry.

[13] Santhanam-Martin R, Fraser N, Jenkins A, Tuncer C (2017). Evaluation of cultural responsiveness using a transcultural secondary consultation model. Transcult Psychiatry.

[14] Fernando S (2005). Multicultural mental health services: projects for minority ethnic communities in England. Transcult Psychiatry.

[15] Horlings A, Hein I (2018). Psychiatric screening and interventions for minor refugees in Europe: an overview of approaches and tools. Eur J Pediatr.

[16] Onyut LP, Neuner F, Schauer E, Ertl V, Odenwald M, Schauer M (2005). Narrative exposure therapy as a treatment for child war survivors with posttraumatic stress disorder: two case reports and a pilot study in an African refugee settlement. BMC Psychiatry.

[17] Oras R, de Ezpeleta SC, Ahmad A (2004). Treatment of traumatized refugee children with eye movement desensitization and reprocessing in a psychodynamic context. Nord J Psychiatry.

[18] Unterhitzenberger J, Eberle-Sejari R, Rassenhofer M, Sukale T, Rosner R, Goldbeck L (2015). Trauma-focused cognitive behavioral therapy with unaccompanied refugee minors: a case series. BMC Psychiatry.

[19] Gesine S, Felicia H, Rose MM (2009). Transcultural clinical work with immigrants, asylum seekers and refugees at Avicenne hospital, France. Int J Migr Heal Soc Care.

[20] Devereux G (1967). From anxiety to method in the behavioral sciences. Reprint 2014.

[21] Nathan T (1986). La folie des autres.

[22] Baubet T, Moro MR (2009). Psychopathologie transculturelle.

[23] Luborsky L, Rosenthal R, Diguer L, Andrusyna TP, Berman JS, Levitt JT (2002). The dodo bird verdict is alive and well—mostly. Clin Psychol Sci Pract.

[24] Lambert MJ, Barley DE (2001). Research summary on the therapeutic relationship and psychotherapy outcome. Psychother Theory Res Pract Train.

[25] Cuijpers P, Reijnders M, Huibers MJH (2019). The role of common factors in psychotherapy outcomes. Annu Rev Clin Psychol.

[26] Murphy R, Hutton P (2018). Practitioner review: therapist variability, patient-reported therapeutic alliance, and clinical outcomes in adolescents undergoing mental health treatment – a systematic review and meta-analysis. J Child Psychol Psychiatry.

[27] Kazdin AE, Marciano PL, Whitley MK (2005). The therapeutic alliance in cognitive-behavioral treatment of children referred for oppositional, aggressive, and antisocial behavior. J Consult Clin Psychol.

[28] Moro MR (1998). Psychothérapie transculturelle des enfants de migrants.

[29] Moro MR (2014). Parenthood in migration: how to face vulnerability. Cult Med Psychiatry.

[30] Lachal J, Moro M-R (2019). Assessment of transcultural psychotherapy in child major depressive disorder.

[31] Truog RD (2012). Patients and doctors — the evolution of a relationship. N Engl J Med.

[32] Hayes D, Edbrooke-Childs J, Town R, Wolpert M, Midgley N (2019). Barriers and facilitators to shared decision making in child and youth mental health: clinician perspectives using the theoretical domains framework. Eur Child Adolesc Psychiatry.

[33] Deshpande P, Bl S, Rajan S, Abdul Nazir C (2011). Patient-reported outcomes: a new era in clinical research. Perspect Clin Res.

[34] APA Presidential Task Force on Evidence-Based Practice (2006). Evidence-based practice in psychology. Am Psychol.

[35] Hill CE, Chui H, Baumann E (2013). Revisiting and reenvisioning the outcome problem in psychotherapy: an argument to include individualized and qualitative measurement. Psychotherapy.

[36] Sibeoni J, Verneuil L, Manolios E, Révah-Levy A (2020). A specific method for qualitative medical research: the IPSE (inductive process to analyze the structure of lived experience) approach. BMC Med Res Methodol.

[37] Elliott R. Qualitative methods for studying psychotherapy change processes. In: Harper D, Thompson A, éditeurs. Qualitative research methods in mental health & psychotherapy. Chichester: Wiley-Blackwell; 2011 [cité 3 avr 2019]. p. 69-81. Disponible sur: https://strathprints.strath.ac.uk/42662/.

[38] Carretier E, Grau L, Mansouri M, Moro MR, Lachal J (2020). Qualitative assessment of transcultural psychotherapy by adolescents and their migrant families: subjective experience and perceived effectiveness. PLoS One.

[39] Patton M (2002). Qualitative research and evaluation methods, 3rd éd.

[40] Smith JA, Osborn M (2003). Interpretative phenomenological analysis. Qualitative psychology: a practical guide to research methods.

[41] Husserl E (1977). Cartesian meditations : an introduction to phenomenology.

[42] Lachal J, Simon A, Hassler C, Barry C, Camara H, Massari N (2020). Epidemiological description of 529 families referred for French transcultural psychotherapy: a decade of experience. PLoS One.

[43] Barrett MS, Berman JS (2001). Is psychotherapy more effective when therapists disclose information about themselves?. J Consult Clin Psychol.

[44] Knox S, Hill CE (2003). Therapist self-disclosure: research-based suggestions for practitioners. J Clin Psychol.

[45] Goldfried MR, Burckell LA, Eubanks-Carter C (2003). Therapist self-disclosure in cognitive-behavior therapy. J Clin Psychol.

[46] Simonds LM, Spokes N (2017). Therapist self-disclosure and the therapeutic alliance in the treatment of eating problems. Eat Disord.

[47] Brown JR, Holloway ED, Akakpo TF, Aalsma MC (2014). « straight up »: enhancing rapport and therapeutic alliance with previously-detained youth in the delivery of mental health services. Community Ment Health J.

[48] Winnicott DW. The child and the family: first relationships. London: Tavistock publications; 1957. p. 147.

[49] Bion W (1961). Experiences in groups.

[50] Kaës R (2007). The question of the unconscious in common and shared psychic spaces. The unconscious: further reflections.

[51] Radjack R, Rizzi AT, Harf A, Moro MR (2017). Actualités en psychiatrie transculturelle en France. Annales Médico-psychologiques, revue psychiatrique.

[52] Zempléni A (1983). Le Sens de l’insensé : de l’interprétation « magico-religieuse » des troubles psychiques. Psychiatr Fr.

[53] Lecomte G, Castonguay LG (1987). Rapprochement et intégration en psychothérapie: psychanalyse, behaviorisme et humanisme.

[54] Diorinou M, Tseliou E (2014). Studying circular questioning « in situ »: discourse analysis of a first systemic family therapy session. J Marital Fam Ther.

[55] Altimir C, Capella C, Núñez L, Abarzúa M, Krause M (2017). Meeting in difference: revisiting the therapeutic relationship based on patients′ and therapists′ experiences in several clinical contexts. J Clin Psychol.

[56] Hawley KM, Weisz JR (2005). Youth versus parent working alliance in usual clinical care: distinctive associations with retention, satisfaction, and treatment outcome. J Clin Child Adolesc Psychol.

[57] Kivlighan DM, Arthur EG (2000). Convergence in client and counselor recall of important session events. J Couns Psychol.

